# Three-dimensional stereophotogrammetry vs. standard measurements for soft tissue and dental arches analysis in cleft lip and palate: Systematic review

**DOI:** 10.4317/medoral.28057

**Published:** 2026-03-07

**Authors:** Victor Fabrizio Cabrera Pazmiño, Joel Ferreira Santiago Junior, Simone Soares

**Affiliations:** 1 Universidad Espíritu Santo, Samborondón, Ecuador; 2 Department of Dental Materials and Prosthetics, School of Dentistry of Ribeirão Preto, University of São Paulo (FORP-USP); 3 Department of Prosthodontics and Periodontology, Bauru Dental School, FOB and Hospital for Rehabilitation of Craniofacial Anomalies, HRAC of the University of Sao Paulo, USP, Bauru. SP, Brazil

## Abstract

**Background:**

Cleft lip and palate (CLP) are the most prevalent congenital anomalies, often resulting in impaired maxillofacial complex in all three planes of space and maxillary growth deficiency, leading to aesthetic and functional challenges. Traditional methods like calipers and rulers have limitations in precision, reproducibility, and volumetric capture for soft tissues (ST) and dental arches (DA) in CLP patients. Three-Dimensional Stereophotogrammetry (3DS) offers a non-invasive digital alternative for accurate 3D imaging. The aim of this systematic review of comparative studies was to evaluate 3DS versus standard measurements (SM) for ST and DA analysis in CLP.

**Material and Methods:**

This study identified comparative studies in humans. Six electronic databases were searched to find articles meeting the eligibility criteria up to December 2025, without language or date restrictions. Risk of bias for each included study was assessed using the 4-stage quality assessment tool for diagnostic accuracy studies (QUADAS-2). Data on outcomes of interest were extracted and tabulated. Due to methodological heterogeneity, a qualitative synthesis was performed, with quantitative evaluation where possible; no meta-analysis was conducted.

**Results:**

From 788 records (413 PubMed, 150 ScienceDirect, 47 Scopus, 34 Web of Science, 8 Cochrane, 136 manual/gray literature), 85 underwent full-text review, yielding 7 studies (269 patients, mean age 12 years, primarily unilateral CLP). Despite methodological heterogeneity among studies, 3DS marked different landmarks in the cleft area (CA) and DA models. There was individual consensus on the accuracy and reproducibility of 3DS markings compared to other methods, and all studies showed low risk of bias in applicability and no meta-analysis due to variability.

**Conclusions:**

All included studies demonstrated that, despite minor methodological differences, digital measurements using 3DS in patients with unilateral cleft lip and palate (UCLP) were more accurate and highly reliable than SM. Future standardized studies are needed for meta-analysis.

## Introduction

Cleft lip and palate (CLP) are the most prevalent congenital anomalies. In this context, studying craniofacial anomalies is essential for developing more accurate treatment techniques to quantify dimensional changes in the face (1).

The maxillofacial complex is impaired in all three planes of space in CLP patients, and maxillary growth deficiency is common, resulting in aesthetic and functional challenges from birth (2 , 3). These challenges vary in degree depending on changes in facial pattern and the severity of the cleft area (CA) (4). Studies have shown that the dimensions of the dental arches (DA) and affected soft tissues (ST) are significantly smaller in patients with CLP compared to non-cleft patients (5 - 7).

Thus, a common approach to studying the effects of different diagnostic protocols and quantifying the actual extent of alveolar and palatal defects involves assessing the dimensions of the CA using direct measurements (DM), 2D measurements (2DM), 2D photography (2DP), calipers/pachymeter measurements on models (CM), and plaster models (PM) (8). However, the literature has shown that these methods can lead to errors not only during patient positioning, transportation, and storage of documentation (9), but also to omissions of information and significant differences between operators in measurements of ST, bone, and alveolar defects (10).

In light of this, methods such as volumetric analysis using three-dimensional (3D) digital imaging via Three-Dimensional stereophotogrammetry (3DS) have been successfully applied, demonstrating accuracy, fidelity, and rapid recording in individuals with CLP (11 , 12). Thus, several studies have shown that 3D imaging is clinically acceptable and reproducible compared to other diagnostic methods (13 , 14).

Therefore, analyzing digital models in association with patients' facial characteristics has enriched the individualization of diagnosis, planning, and treatment execution (15), it also facilitates three-dimensional calculations of the CA in CLP patients, which would be impossible with other methods (16).

Nevertheless, the body of evidence comparing diagnostic methods in CLP patients remains limited, with small samples, varying software, and non-standardized methodologies. Therefore, the purpose of this systematic review was to evaluate the reliability of three-dimensional stereophotogrammetry versus standard measurements (SM) for analyzing ST and DA in CLP.

## Material and Methods

The protocol for this review was registered in the International Prospective Register of Systematic Reviews (PROSPERO: CRD42021261518) and conducted in accordance with the PRISMA 2020 guidelines.

Clinical scenarios of interest

CLP patients who underwent analysis using 3DS and other diagnostic methods for hard and ST to quantify dimensional changes.

Eligibility Criteria

The central clinical question of this systematic review was formatted according to the PICO (Population, Intervention, Comparison, and Outcomes) framework for evidence-based practice (17): Population: CLP patients; Intervention: Analysis of CLP using 3DS; Comparison: Analysis of CLP using other measurement and diagnostic methods; Outcome: Evaluation of the fidelity of data obtained from 3DS analysis of hard and ST compared to other diagnostic methods.

Inclusion and exclusion criteria

The inclusion criteria were: Studies published in English; randomized controlled trials, prospective and retrospective studies with comparative methodology, and comparative studies; studies comparing at least two analysis methods (3DS versus SM); and papers involving patients with CLP who underwent analysis methods to quantify dimensional changes in hard and ST of the face and upper arch (3DS versus SM).

The exclusion criteria were as follows: Case reports and case series; literature reviews; non-comparative studies; experimental animal studies and studies involving syndromic patients.

Information Sources and Literature Search Protocol

Six electronic databases were searched to identify articles that met the eligibility criteria: PubMed (National Library of Medicine), Science Direct (Elsevier), Scopus (Elsevier), Web of Science (Clarivate Analytics), LILACS (Literatura Latinoamericana y del Caribe en Ciencias de la Salud), and Cochrane (Cochrane Central Register of Controlled Trials). There were no restrictions on the year of publication for included studies. The search was conducted from December 2019 to December 2025.

The search strategy combined MeSH (Medical Subject Headings) terms and English keywords, linked by Boolean operators (AND, OR, NOT). Due to variations in controlled vocabulary and syntactic limitations across databases, search strategies were adapted individually for each database; however, the core concepts, terms, and Boolean logic remained consistent, as detailed in Table 1.

Table 1To complement the electronic search, a manual search was performed in relevant scientific journals using the descriptors. The journals included: The Cleft Palate-Craniofacial Journal, Journal of Cleft Lip Palate and Craniofacial Anomalies, Neonatal Cleft Lip and Cleft Palate Repair, International Journal of Anesthesiology &amp; Pain Medicine, Journal of Universal Surgery, British Dental Journal, American Cleft Palate-Craniofacial Association, Journal of Applied Oral Science, Oral Health Case Reports, Oral Surgery, Oral Medicine, Oral Pathology and Oral Radiology, Surgery, International Journal of Oral &amp; Maxillofacial Surgery, Birth Defects Research, and Journal of Cranio-Maxillo-Facial Surgery.

Additionally, a search of the gray literature was conducted in sources such as Google Scholar, ProQuest, and OpenGrey.

Study selection

All titles and abstracts identified in the literature searches were independently screened by two calibrated reviewers (V.F.C.P. and J.F.S.J.). The reviewers applied the eligibility criteria during the full-text assessment of potentially eligible studies. Subsequently, the final selection of articles for qualitative and/or quantitative analysis was performed. In case of disagreements during the final selection of a study, the reviewers resolved them through open discussion with another co-author (S.S.).

Risk Assessment

The risk of bias for each included study was assessed using the four-domain quality assessment tool for diagnostic accuracy studies (QUADAS-2) (18). The two reviewers (V.F.C.P. and J.F.S.J.) independently scored each item as 'high,' 'low,' or 'uncertain' and assessed the quality of each study. The four methodological domains assessed were patient selection, index test, reference standard, and flow and timing. Review Manager 5.4 software (RevMan 5.4, The Nordic Cochrane Centre, The Cochrane Collaboration, Copenhagen, Denmark) was used to generate the risk-of-bias graphs and summary.

Data synthesis and summary of findings

The data were organized in evidence tables, and a descriptive summary was created to examine the quantity of data and variations among studies (including study characteristics, quality, and outcomes). All types of outcome measurements were considered, including landmark positions, linear measurements, reference points and lines, distances, and software used for model analysis.

Variable outcomes

The primary outcome was the evaluation of the reliability of data obtained from 3DS analysis in patients with CLP compared to SM. The secondary outcome was the evaluation and comparison of hard and soft facial tissues in patients with CLP. Figures were generated from data extracted using RevMan 5 (https://training.cochrane.org/online-learning/core-software/revman).

## Results

Literature Selection Process

**Figure 1:**
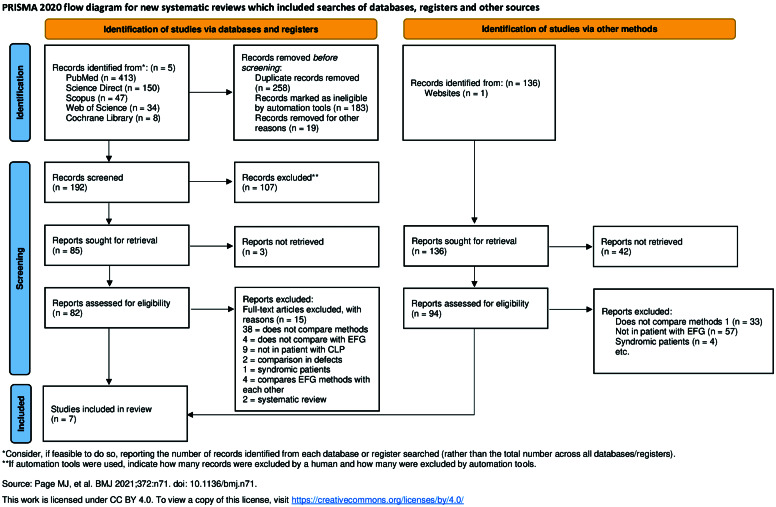
PRISMA 2020 flow diagram of the selection process for articles found in the selected databases. Source: https//www.prisma-statement.org/.

The initial search yielded a total of 788 articles: 413 from PubMed, 150 from Science Direct, 47 from Scopus, 34 from Web of Science, and 8 from the Cochrane Library. An additional 136 articles were identified through manual searching. After title and abstract screening, 85 articles were selected for full-text review. Of these, 78 were excluded after full-text assessment, with reasons summarized in Figure 1. Thus, 7 articles were finally included (19 - 25). The entire article selection process is illustrated in Figure 1.

1

Risk of bias in the studies

**Figure 2:**
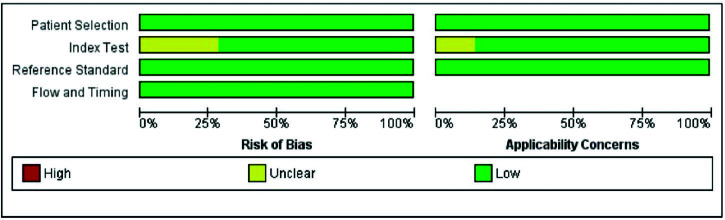
Results of QUADAS-2 Quality Assessment of Studies - A) Risk of bias graph.

**Figure 3:**
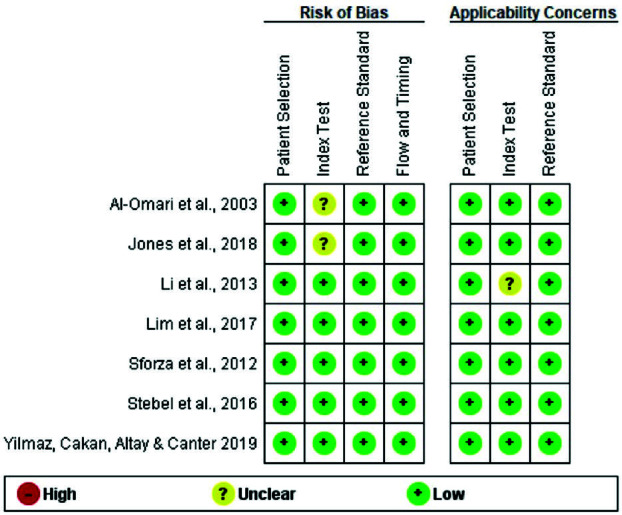
Results of QUADAS-2 Quality Assessment of Studies - B) Risk of bias summary.

Although the studies' methodologies were highly heterogeneous, all demonstrated a low risk of bias in terms of applicability. In all studies, domain 1 (patient selection) was rated as low risk of bias (Figure 2). In domain 2 (index test), only two studies received a high risk rating due to unclear data interpretation resulting from methodological limitations. Domains 3 (reference standard) and 4 (flow and timing) were rated as low risk in all studies. The overall QUADAS-2 risk-of-bias assessment for each study is presented in Figure 3.

2

3

Due to significant heterogeneity in data, reference points, and measurement approaches across the included studies, a meta-analysis was not warranted. Therefore, only a qualitative synthesis is provided. The seven included studies were comparative and involved a total of 269 patients with unilateral cleft lip and palate (UCLP). These included 138 plaster models (PM), 46 silicone impressions, 10 direct measurements (DM), and over 60 random images. The mean age of the patients was 12 years (Table 2).

**Figure 4:**
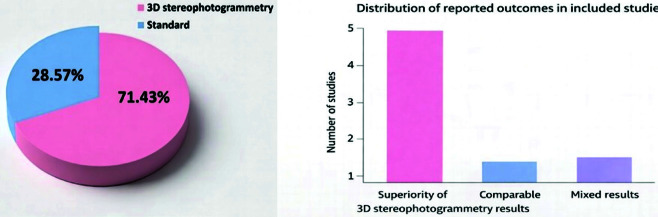
Proportion of studies favoring 3DS over standard methodos/Distribution of reported outcomes in included studies.

Table 2Figure 4 summarizes the overall distribution of evidence among the included studies. It shows that 71.43% of studies reported superior accuracy and reliability of 3D stereophotogrammetry compared to SM methods, while 28.57% reported comparable results. Notably, no study found conventional techniques to be superior, indicating a clear predominance of evidence favoring 3D stereophotogrammetry.

4

It illustrates the qualitative distribution of reported outcomes across the included studies, distinguishing between clear superiority, comparable findings, and mixed results. This breakdown highlights methodological and outcome-related heterogeneity among the studies while confirming the absence of evidence favoring SM methods over 3D stereophotogrammetry (Figure 4).

One study (19) evaluated residual facial deformity in patients with repaired UCLP and compared ratings of facial deformity made by health care professionals with those made by lay assessors, using the reliability of clinical assessment, two-dimensional color transparencies, and 3D images. Two studies (20 , 21), performed the comparisons of the CA using DM and facial PM, with measurements obtained via 3DS On the other hand, studies (22 , 23) performed measurements of the CA and DA using PM and silicone impressions with photography and compared these data with those obtained by 3DS from the same area. Other included studies (24 , 25) comparatively assessed 3DS facial images and 2DP through the marking of different points in the nasolabial region of patients with UCLP (Table 3).

Table 3Al-Omari et al. (2003) evaluated residual cleft-related facial deformity using full-face, lip, nose, and midface views. Five professionals and five laypersons scored the images using a 5-point ordinal scale on two occasions with a 1-month interval. They concluded that the methods showed equivalent reliability in assessing photographs of the entire face, but 2D images had higher reliability in the nasolabial region, whereas 3D images were more reliable in the midface.

Sforza et al. (2012) evaluated plaster models (PM) of the palate in children with unilateral cleft lip and palate (UCLP) using direct anthropometry and compared them to digitized models obtained through 3D stereophotogrammetry (3DS). They reported statistically significant differences (p&lt;0.05) between calipers and 3D measurements for almost all distances analyzed, with mean absolute differences ranging from 0.22 to 3.41 mm. Generally, calipers values were larger than those obtained by 3DS (Table 4).

Table 4Li et al. (2013) compared the accuracy and precision of acquired 3D facial data and direct measurements (DM) in children with UCLP. The results showed no significant differences (p&gt;0.05) in linear measurements between direct anthropometric measurements and 3D image measurements overall. However, the localization of four landmarks was inconsistent in repeated inter-observer reliability tests on preoperative images (p&lt;0.05), whereas intra-observer reliability was good in both pre- and postoperative images (p&gt;0.05) (Table 3).

Lim et al. (2017) compared two-dimensional (2D) and three-dimensional (3D) measurements for the alveolar molding effect in patients with UCLP. The results showed that two angular variables (out of 12 total) differed in statistical interpretation of change after nasoalveolar molding treatment between 2D and 3D measurements. However, Bland-Altman analysis revealed no significant difference in the magnitude of change for these variables between the two measurement methods (Table 4).

Yilmaz Çakan, Altay, and Canter (2019) evaluated the reliability of methods and the influence of knowledge and skill on inter-examiner and intra-examiner nasolabial measurements in plaster models (PM) and 3DS. The results showed that measurements on 3DS images demonstrated higher reliability compared to PM measurements, particularly when performed by a postgraduate student rather than a lecturer (Table 3).

Stebel et al. (2016) compared the reliability of assessing nasal form, nasal deviation, vermilion border, and nasolabial profile on 3D images and 2D photographs (2DP) in prepubertal children with UCLP using a 100-mm analog visual scale (AVS). The results showed higher reliability for assessments on 3D images (0.733-0.857) than on 2D images (0.151-0.611). 3D images were considered more informative than 2D images (p=0.001), but probably more difficult to evaluate (p=0.06).

Jones et al. (2018) determined the reliability of measurements of nasolabial appearance (nasolabial profile [NLP], nasolabial frontal [NLF], and vermilion border aesthetics [VB]) using 3DS images versus 2DP in patients with repaired complete unilateral cleft lip and palate (CUCLP), using the 5-point scale described by Asher-McDade with a modified Q-sort method. Contrary to other studies, this investigation found good inter-rater reliability for 2DP (=0.607-0.710) and fair to good for 3DS (=0.374-0.769). Intraobserver reliability was good to very good for 2DP (=0.749-0.836) and moderate to good for 3DS images (=0.554-0.855).

## Discussion

The degree of accuracy required for any measurement or diagnostic tool should be determined based on its intended application. When comparing a new diagnostic or treatment planning tool with an established gold standard, it is often assumed that the existing tool is highly accurate (26). Direct measurements (DM), two-dimensional measurements (2DM), plaster models (PM), 2D photography (2DP), and other conventional methods are widely accepted as providing an adequate physical representation of the dental arches (DA) in patients. However, when measuring the cleft area (CA) and its complex structures in patients with varying degrees of cleft lip and palate (CLP) using conventional two-dimensional methods, these techniques exhibit random errors in repeated intra-examiner and inter-examiner measurements (23 , 27 , 28).

When considering the quantitative assessment of cleft depth to characterize CLP, we recognize that 3DS, which involves capturing images through multiple simultaneous photographs and is commonly used for facial ST evaluation (29), provides clinically relevant information and accurate data on palatal morphology before, during, and after treatment. This makes 3DS essential for better understanding the real effects of therapeutic protocols in these patients (23). 3DS systems offer high quality for facial morphological examinations and provide several advantages over previous methods, including fast acquisition, improved image size and quality, low cost, and the absence of hazardous procedures. As a result, 3DS has become the primary tool for surface investigations (9 , 30).

In the present systematic review, five of the included articles (Sforza et al., 2012; Li et al., 2013; Stebel et al., 2016; Lim et al., 2017; Ylmaz, Çakan, Altay, &amp; Canter, 2019), regardless of their objectives and methodologies, concluded that the precision and accuracy (reliability and validity) of 3DS measurements were clinically acceptable in terms of comparison and reproducibility with other standard diagnostic methods in patients with UCLP. The exceptions were the studies by Al-Omari et al. (2003) and Jones et al. (2018), which found that reliability does not improve with current 3DS technology. The term "reliability" refers to the overall consistency of a measurement under constant conditions (31). "Reproducibility" refers to the ability of results to agree when the instrument is applied uniformly and repeatedly on invariant objects. "Validity" refers to the degree to which the measurement method adequately captures the true value of what is being measured, observed, or interpreted (32).

The variation in results from the study by Al-Omari et al. (2003) may be attributed to the different ages of the study subjects (10 and 30 years), operator limitations in image manipulation, and the diversity of professionals involved. Several surgeons treated patients with different surgical techniques, which may have increased result variability and caused difficulties in scoring, despite comparing three stimulus media for facial aesthetics evaluation in CLP: Clinical assessment, 2D color transparencies, and 3DS. Additionally, 2D and 3D images were equivalent for aesthetic assessment of the full face, lip, nose, and mid-nose (size, smoothness, visibility of the cleft scar, and continuity of the vermilion border), but not for specific landmarks such as philtrum width, nasolabial points, columella points, nostril, and nose points. Furthermore, this study used an aesthetic index developed by Asher-McDade et al. (1991), whereas the AVS scale is recommended for these measurements (33 , 34).

In the study by Sforza et al. (2012), systematic errors were found for all measurements located on the crest of the alveolar segments when marking reference points on linear distances, which highlights the importance of correct marking and interpretation of points by the examiner. Other studies confirm these differences between the two measurement methods that performed linear measurements between reference points with digital calipers directly on PM (35 , 36).

The accuracy of 3DS measurements in the study by Li et al. (2013) was considered highly reliable; however, statistically significant differences were observed compared to direct anthropometric measurements in most cases. Additionally, there were variations in the identification of specific points on the 3D images by the examiners. This indicates that landmark recognition can be highly subjective, particularly on the cleft side of the 3D model, and suggests that landmarks should be identified by a single experienced person to minimize error in patients with UCLP.

In the study by Lim et al. (2017), evaluation of 2D and 3D measurements at common landmarks and lines using linear and angular variables in different stages of UCLP patients showed no statistically significant differences in the interpretation of points (p &gt; 0.05) at various angles. Similarly, as described in other studies, misinterpretations of 3D measurement results may occur due to inaccurate landmark recognition and the use of incorrect reference systems (37 , 38).

On the other hand, Yilmaz Çakan, Altay, &amp; Canter (2019) showed that the reliability of measurements performed with 3DS depends on the identification of 3D landmarks, the morphology of the anatomical structure of the cleft, image quality, and the examiners' familiarity and experience in handling the software and measurement methods. In 3D facial scans, the identification of landmarks on well-defined edges is better, resulting in higher reproducibility. Furthermore, points located on curved slopes with anatomical variations, as in cases of clefts, are difficult to determine (39). This coincides with another study that emphasizes that the reproducibility of landmark identification in 3D images requires examiner experience (40).

Stebel et al. (2016), in their study of the assessment of nasolabial aesthetics in patients with CLP through intra-examiner analysis of 2D and 3DS photographs, justified the reliability of their measurements with their results. They found that 2D assessment was associated with lower reproducibility than assessment on 3D images. It should also be noted that the manipulation of 3D images was performed in all directions without any temporal restriction. The authors recommend the use of reference photographs together with the AVS or a semi-continuous numerical scale (from 0 to 200) to obtain more reliable results, and they point out that clinical evaluation is the gold standard for assessing nasolabial esthetics. Another important factor was the evaluators' familiarity with 3D images, unlike other studies with lay judges, where operator calibration was conducted over a longer period (41 , 42).

However, Jones et al. (2018) showed that while 3D imaging may offer additional views compared to 2DP, clinical outcomes were more reliably graded using 2DP to assess NLF, NLP, and VB, agreeing with Stebel et al. (2016). It is worth noting that VB assessment was the only measure that showed worse results for 2D imaging. Another factor to highlight is the difficulty of downloading and sharing 2D images compared to 3DS systems, which facilitate standardization of measurements.

The use of direct measurements (DM), two-dimensional measurements (2DM), and plaster models (PM) as aids in the study and diagnosis of patients with CLP has been employed for many years. However, their measurement, production, and elaboration are always associated with the disadvantage of ST deformation caused by impression materials and the accuracy of these procedures by the examiner, which can influence treatment outcomes (43). Fleming et al. (2011), in their systematic review, compared the reliability of measurements performed from PM and DM of the dental arches (DA) and concluded that the use of DM is recommended as an excellent alternative compared to other methods (44).

Most authors highlight the limited number of studies that have examined the reliability of 3DS technology for assessing patients with CLP. We believe that, although there are well-defined treatment protocols for the care of oral clefts from the prenatal period to adulthood, there is a lack of studies developing a standardized methodology capable of accurately determining the volumetric size of bone defects and ST through volumetric imaging examinations, especially in standardizing the reference points to be examined. Such surface analysis research would facilitate the reproducibility of measurements and allow for better therapeutic outcomes in patients with UCLP.

This paper provides important evidence that 3D scanning of facial structures affected by CLP is a reliable alternative for the analysis of craniofacial deformities.

Limitations

Several methodological limitations of this review should be considered. First, there was no standardization of methodology across the included studies. Second, there was large variation in sample sizes across the studies; for example, Al-Omari et al. (2003) evaluated 31 patients, Sforza et al. (2012) evaluated 96 models, and Ylmaz, Çakan, Altay, &amp; Canter (2019) had a sample size of 42 models. Similarly, Li et al. (2013) evaluated 10 patients, Lim et al. (2017) studied a total of 23 patients, Stebel et al. (2016) examined 40 patients, and Jones et al. (2018) evaluated 27 patients. Third, the studies by Li et al. (2013) and Ylmaz, Çakan, Altay, &amp; Canter (2019) quantitatively evaluated comparisons of data on cleft lip and palate, while Lim et al. (2017) and Sforza et al. (2012) focused on comparisons of data on dental arches (DA). Although Al-Omari et al. (2003), Stebel et al. (2016), and Jones et al. (2018) compared 2D photographs versus 3DS images, the reference points for markings and measurements were different, making comparisons between them unfeasible. The same reference points, software, angular and linear measurements in the cleft area (CA) and nasopalatine region were not considered, and the diagnostic processes were unrelated to the intra- and extra-arch measurements.

## Conclusions

Within the limits of this study, we conclude that all included studies demonstrated that digital measurements with 3DS in patients with CLP were more accurate and highly reliable compared to other standard analysis methods, despite some minor methodological differences. Therefore, digital measurements with 3DS as a diagnostic aid is an appropriate method for measuring facial tissues. However, the limited number of papers and the heterogeneity of the studies did not allow for a meta-analysis. Therefore, we recommend developing more studies with similar methodologies to standardize clinical diagnostic protocols for these patients.

## Figures and Tables

**Table 1:** Search strings entered into the search tools and databases. The number of items retrieved (N) is shown in this table.

Database	Descriptors/Strategy	N
PubMed/MEDLINE	Randomized Controlled Trial OR Randomized Control Trial OR Randomized Clinical Study OR Randomized Clinical Trial OR Comparative Study OR Comparative Trial OR Prospective OR Retrospective) AND (3D images OR 3D digital stereophotogrammetry OR Three Dimensional Images OR Stereophotogrammetry OR Photogrammetries OR Photogrammetric OR Photogrammetry OR Radiostereometric Analysis OR Stereophotogrammetries OR stereophotogrammetrists OR Imaging Diagnostic OR Radiostereometry) AND (Dental Arches OR Maxillary OR Maxilla OR Bone Tissues OR Hard Tissues OR Soft Tissues) AND (Cleft Lip and Palate OR Unilateral cleft lip and palate OR Bilateral cleft lip and palate OR Complete Cleft Lip and Palate OR Orofacial Cleft OR Cleft lip with or without cleft palate) NOT (Case Series OR Case Reports OR Systematic Review OR Meta-analysis)	#413
ScienceDirect	(Randomized Control Trial OR Comparative Study OR Prospective OR Retrospective) AND (Stereophotogrammetry OR Stereophotogrammetries) AND (Dental Arches OR Maxillary OR Maxilla) AND (Cleft lip and palate OR Orofacial cleft)	#150
Scopus	Randomized OR comparative OR prospective OR retrospective AND stereophotogrammetry OR stereophotogrammetry AND dental AND arches OR maxilla AND cleft AND lip AND palate OR orofacial AND cleft	#47
Web of Science	(Randomized Controlled Trial OR Randomized Control Trial OR Randomized Clinical Study OR Randomized Clinical Trial OR Comparative Study OR Comparative Trial OR Prospective OR Retrospective) AND (3D images OR 3D digital stereophotogrammetry OR Three Dimensional Images OR Stereophotogrammetry OR Photogrammetries OR Photogrammetric OR Photogrammetry OR Radiostereometric Analysis OR Stereophotogrammetries OR stereophotogrammetrists OR Imaging Diagnostic OR Radiostereometry) AND (Dental Arches OR Maxillary OR Maxilla OR Bone Tissues OR Hard Tissues OR Soft Tissues) AND (Cleft Lip and Palate OR Unilateral cleft lip and palate OR Bilateral cleft lip and palate OR Complete Cleft Lip and Palate OR Orofacial Cleft OR Cleft lip with or without cleft palate) NOT (Case Series OR Case Reports OR Systematic Review OR Meta-analysis)	#34
Cochrane Library	(Randomized Controlled Trial OR Comparative Study OR Comparative Trial OR Prospective Study OR Retrospective Study) AND (3D digital stereophotogrammetry OR Three Dimensional Images OR Stereophotogrammetry OR Photogrammetries OR Photogrammetric OR Photogrammetry OR Stereophotogrammetries OR stereophotogrammetrists OR Imaging Diagnostic) AND (Dental Arches OR Maxillary OR Maxilla) AND (Cleft Lip and Palate OR Orofacial Cleft) NOT (Case Series OR Case Reports OR Systematic Review OR Meta-analysis)	#8
Google Scholar	(Randomized Control Trial OR Comparative Study OR Prospective OR Retrospective) AND (Stereophotogrammetry OR Stereophotogrammetries) AND (Dental Arches OR Maxillary OR Maxilla) AND (Cleft lip and palate OR Orofacial cleft) NOT (Case Series OR Case Reports)	#136

1

**Table 2:** Qualitative table of the general design of the included studies.

­Study	Kind Of Study	Year	Country	Patients and Gender (N)	MeanAge	Scanned Area	Methodological Comparisons	Program Used	Data Standardization	Scanning of the Gypsum Model/Model Analysis	Position For Taking Pictures (Children)	Data storage format	Number of Measurements/Repeats in a Session/Pictures
Al-Omari and cols.	Comparative	2003	Sweden	31 (21m and 10f)	20.5 years	The relation of the nose and the upper lip to the forehead and lower face	2D photographs	NR	NR	NR	Subjects tilted the head back to bring the alar domes to a level below the eyebrows but above the canthi	NR	Two times/Interval 1 week
							C3D stereophotogrammetry machine (3D Matic Faraday Partnership, Glasgow, Scotland)	C3D software	GL viewer program (EMD Enterprise, Iowa City, Iowa)	NR	Maintain a natural facial expression with lips held gently together and the head tilted back slightly to view the nostril outlines from below	NR	Two times/Interval 1 week
Sforza and cols.	Comparative	2012	Italia sample from Colombia	96 (models)	Neonatal	Palate gypsum models on dental arches	Calipers on gypsum models	NR	NR	NR	NR	NR	Twice
							Stereophotogrammetry	Stereophotogrammetry system 3D (VECTRA-3D; Canfield Scientific, Inc., Fairfield, NJ)	Vectra´s image processing software	NR	NR	NR	Twice
Li and cols.	Comparative	2013	China	10 (6m and 4f)	3-3,5 months	Nasal area and the upper lip to the commissures on the face	Direct measurement (clinical calipers and calibrated calipers)	NR	NR	NR	NR	NR	5 (c/72h)
							3DSS II, Shanghai Digital Manufacturing Corporation, Shanghai, China	NR	Geomagic Studio 10.0 software (Geomagic Inc., Research Gtriangle Park, NC, USA)	NR	Frankfort plan 10º-20º	ASCII files	10 (preoperative) 10 (postoperative)
Stebeland cols.	Comparative	2016	Switzerland	40	10 years	Nasolabial area	2D photographs	NR	PowerPoint	NR	Positioned in the natural head position and asked to keep their eyes open and to relax their facial musculature	NR	3 weeks
							3D stereophotogrammetric images of the face	3dMD face System; 3D LLC, Atlanta, Georgia, USA	3D viewer (3D-Tool, version 9, 3D-Tool, Weinheim, Germany)	NR		NR	3 weeks
Lim and cols.	Comparative	2017	South Korea	23(10m-13f) 46 (models)	23,5 days	Alveolar zone of the maxilla in dental arches	2D measurements/Molding Silicone (GC America Inc Alsip, IL) and photographic pictures (Nikon D70)	Scanner (EPSON Perfection) Software the analize the model (V-ceph 4.0, CyberMed Co, Seul, Korea)	NR	NR	NR	NR	2 times/Interval 2 weeks
							Laser scanner (Orapix, Seul, Korea)	3D software )3Txer, Seul, Korea=	NR	V-ceph 4.0, CyberMed Co., Seul, Korea	NR	NR	2 times/Interval 2 weeks
Jones and cols.	Comparative	2018	Canada	27 (21m and 6f)	6 years and 11 months	Nasolabial	2D photographs	NR	Microsoft PowerPoint 2007	NR	Frontal and profile	Individual files on several identical CD-ROM disks	Twice, on separate days
							3D stereophotogrammetric facial surface image	3dMD Face System, Atlanta, GA; model manufactured February 2006	3dMD software	NR			Twice, on separate days
Yilmaz and cols.	Comparative	2019	Turkey	42	NR	Baby phase model	Gypsum models	Pachymeter Opto-Rs 232 simplex/dÃºplex, Sylvac/Fowler, Crissier, Switzerland.	NR	NR	NR	NR	1 time after 3 weeks
							3dMDface system (3dMD, Atlanta, GA)	3dMD software program (3dMD, Atlanta, GA)	NR	NR	NR	NR	1 time after

NR: Not Reported.

**Table 3:** Quantitative table of the comparison of data from CLP studies.

	Studies									
	Li et al., 2013		Yilmaz et al., 2018		Stebel et al., 2016		Jones et al., 2018		Al-Omari et al., 2003	
Reference points/Methodological comparison (mean)	2D measurements (direct) (mm)	3D measurements (Stereophotogrammetry) (mm)	2D measurements (direct) (mm)	3D measurements (Stereophotogrammetry) (mm)	2D measurements (direct) (mm)	3D measurements (Stereophotogrammetry) (mm)	2D measurements (direct) (mm)	3D measurements (Stereophotogrammetry) (mm)	2D measurements (direct) (mm)	3D measurements (Stereophotogrammetry) (mm)
Philtrum width (non-slit side)	NR	NR	0.814	0.947	NR	NR	NR	NR	NR	NR
Average Philtrum height	NR	NR	0.929	0.970	NR	NR	NR	NR	NR	NR
Right side Philtrum height (non-slotted side)	NR	NR	0.955	0.996	NR	NR	NR	NR	NR	NR
Left side Philtrum height (slit side)	NR	NR	0.977	0.999	NR	NR	NR	NR	NR	NR
Right lip height (non-cleft side)	NR	NR	0.927	0.994	NR	NR	NR	NR	NR	NR
Left lip height (cleft side)	NR	NR	0.957	0.969	NR	NR	NR	NR	NR	NR
Nasal tip protrusion	NR	NR	0.961	0.995	NR	NR	NR	NR	NR	NR
Nasal width	NR	NR	0.988	0.509	NR	NR	NR	NR	NR	NR
Nasal shape	NR	NR	NR	NR	0.869	0.855	NR	NR	NR	NR
Nasal deviation	NR	NR	NR	NR	0.755	0.826	NR	NR	NR	NR
Nasolabial frontal	NR	NR	NR	NR	NR	NR	0.770	0.812	NR	NR
Nasolabial profile	NR	NR	NR	NR	NR	NR	0.678	0.464	NR	NR
Columella length sn0-c0	3.28	3.20	NR	NR	NR	NR	NR	NR	NR	NR
Columella length sn0-c0 (slit side)	1.89	1.75	NR	NR	NR	NR	NR	NR	NR	NR
Sbal-sn nostril width (non-slit side)	6.84	7.75	0.904	0.748	NR	NR	NR	NR	NR	NR
Sbal-sn nostril (slit side)	14.41	15.56	0.984	0.89	NR	NR	NR	NR	NR	NR
Distance from subnasal alar base ac-sn (non-slit side)	16.05	14.79	NR	NR	NR	NR	NR	NR	NR	NR
Distance from subnasal alar base ac-sn (slit side)	20.73	19.34	NR	NR	NR	NR	NR	NR	NR	NR
Alar length ac-prn (non-slotted side)	12.84	13.45	NR	NR	NR	NR	NR	NR	NR	NR
Alar length ac-prn (slit side)	16.76	18.06	NR	NR	NR	NR	NR	NR	NR	NR
Lateral height os upper lip sbal-ch (non-cleft side)	14.51	15.18	NR	NR	NR	NR	NR	NR	NR	NR
Lateral height of upper lip sbal-ch (cleft side)	14.51	15.18	NR	NR	NR	NR	NR	NR	NR	NR
Upper lip height sbal-cph (non-cleft side)	11.26	10.42	NR	NR	NR	NR	NR	NR	NR	NR
Upper lip height sbal-cph (cleft side)	8.79	7.91	NR	NR	NR	NR	NR	NR	NR	NR
Lateral lip length ch-cph (non-cleft side)	15.68	15.99	NR	NR	NR	NR	NR	NR	NR	NR
Lateral lip length ch-cph (cleft side)	11.66	12.79	NR	NR	NR	NR	NR	NR	NR	NR
Vermilion border	NR	NR	NR	NR	0.868	0.869	0.768	0.527	NR	NR
Profile view	NR	NR	NR	NR	0.940	0.889	NR	NR	NR	NR
Cleft lip	NR	NR	0.976	0.997	NR	NR	NR	NR	NR	NR
Nostril height (non-slit side)	NR	NR	0.946	0.990	NR	NR	NR	NR	NR	NR
Nostril height (slit side)	NR	NR	0.834	0.997	NR	NR	NR	NR	NR	NR
Medial nostril height (non-slit side)	NR	NR	0.895	0.929	NR	NR	NR	NR	NR	NR
Medial nostril height (slit side)	NR	NR	0.804	0.966	NR	NR	NR	NR	NR	NR
Lateral nostril height (non-slit side)	NR	NR	0.920	0.982	NR	NR	NR	NR	NR	NR
Lateral nostril height (slit side)	NR	NR	0.989	0.987	NR	NR	NR	NR	NR	NR
Nostril diameter (non-slit side)	NR	NR	0.898	0.996	NR	NR	NR	NR	NR	NR
Nostril diameter (slit side)	NR	NR	0.872	0.998	NR	NR	NR	NR	NR	NR
Full face	NR	NR	NR	NR	NR	NR	NR	NR	0.572	-0.172
Lip	NR	NR	NR	NR	NR	NR	NR	NR	0.325	-0.207
Nose	NR	NR	NR	NR	NR	NR	NR	NR	0.065	-0.161
Midface	NR	NR	NR	NR	NR	NR	NR	NR	0.022	-0.318

NR: Not Reported.

**Table 4:** Quantitative table of the comparison of data from dental arches studies.

	Studies			
	Lim et al., 2016		Sforza et al., 2012	
Reference points/Methodological comparisons (mean)	2D measurements(direct) (mm)	3D measurements (Stereophotogrammetry)(mm)	2D measurements(gypsum models)(mm)	3D measurements (Stereophotogrammetry)(mm)
(PG-PL)	33,77	33,07	NR	NR
(BG-BL)	29,5	30,49	NR	NR
(Trans ACG-ACL)	7,04	7,79	NR	NR
(Sagital ACG-ACL)	5,38	5,55	NR	NR
(Distance ACG-ACL)	9,09	9,95	NR	NR
(MG-(PG-PL)	26,31	27,09	NR	NR
(M L-(PG-PL)	18,39	18,59	NR	NR
(ACG-PG) - (PG-PL)	50,51	55,07	NR	NR
(ACL-PL) - (PG-PL)	66,79	63,01	NR	NR
(ACG-BG-PG)	121,05	108,54	NR	NR
)ACL-BL-PL)	130,92	112,27	NR	NR
(BG-ACG) - (BL-ACL)	123,96	103,87	NR	NR
(Ag-Am)	NR	NR	7.10	7.44
(Cg-Cm)	NR	NR	21.85	21.73
(Pg-Pm)	NR	NR	31.76	31.62
(Ag-Pg)	NR	NR	27.13	30.19
(Am-Pm)	NR	NR	25.60	22.00
(Dm-Cm)	NR	NR	14.80	13.84
(Dg-Cg)	NR	NR	15.33	15.10

NR: Not Reported.

## Data Availability

Declared none.
